# RUNX1-FPDMM in families with mild thrombocytopenia and platelet function anomalies: a case series

**DOI:** 10.3389/fmed.2025.1657054

**Published:** 2025-12-11

**Authors:** Hannah Glonnegger, Doris Boeckelmann, Rebekka Wiedenhöfer, Wolf-Achim Hassenpflug, Tim Ripperger, Dirk Lebrecht, Ralf Knöfler, Oliver Tiebel, Udo Koehler, Claudia Wehr, Harry Sirb, Monika Sparber-Sauer, Katrin Reinsberger, Ayami Yoshimi, Brigitte Strahm, Barbara Zieger

**Affiliations:** 1 Department of Pediatric Hematology, Oncology and Stem Cell Transplantation, Children’s Hospital, Medical Center-University of Freiburg, Faculty of Medicine, University of Freiburg, Freiburg, Germany; 2 Department of Pediatric Hematology and Oncology, University Hospital Eppendorf, Hamburg, Germany; 3 Department of Human Genetics, Hannover Medical School, Hannover, Germany; 4 Department of Pediatric Hemostaseology, Medical Faculty Carl Gustav Carus, Technical University Dresden, Children's Hospital, Dresden, Sachsen, Germany; 5 MGZ – Medical Genetics Center, Munich, Germany; 6 Department of Medicine I/Hematology, Oncology and Stem Cell Transplantation, Medical Center-University of Freiburg, Faculty of Medicine, University of Freiburg, Freiburg, Germany; 7 Medical Center (MVZ) Meerane, Rudolf Virchow Hospital Glauchau, Glauchau, Germany; 8 Department of Pediatric Hematology and Oncology, University Children’s Hospital Tuebingen, Tuebingen, Germany; 9 Stuttgart Cancer Center, Zentrum für Kinder-, Jugend- und Frauenmedizin (Olgahospital), Pädiatrie 5 (Pädiatrische Onkologie, Hämatologie, Immunologie), Klinikum der Landeshauptstadt Stuttgart, Stuttgart, Germany

**Keywords:** RUNX1, FPDMM, platelet granule secretion, thrombocytopenia, thrombocytopathy, predisposition

## Abstract

**Background:**

RUNX1-familial platelet disorder with associated myeloid malignancy (RUNX1-FPDMM) is caused by heterozygous germline variants of *RUNX1*. With the broader application of next-generation sequencing (NGS)-based gene panel analysis in individuals presenting with benign hematologic abnormalities such as thrombocytopenia, pathogenic *RUNX1* variants were more frequently identified, independent of a hematologic malignancy.

**Objective:**

This study aimed to describe the clinical and genetic characteristics of individuals with pathogenic germline *RUNX1* variants, with a particular focus on platelet function and diagnostic challenges.

**Methods:**

We retrospectively analyzed 10 individuals from 6 families with genetically confirmed RUNX1-FPDMM. Platelet counts and function were evaluated using light transmission aggregometry (LTA) and flow cytometry (FC). For genetic analysis, NGS-based panel sequencing for inherited platelet disorders, Sanger sequencing, karyotyping, fluorescence *in situ* hybridization (FISH), and microarray analysis were performed.

**Results:**

Platelet counts ranged between 40 and 208 G/L. In all six tested individuals, LTA revealed impaired aggregation in response to collagen, adenosine diphosphate (ADP), and epinephrine. FC analysis identified a pronounced granule secretion defect in three of the eight tested individuals. Disease-causing *RUNX1* variants included whole-gene or intragenic deletions, one missense, two not previously reported non-sense variants, and a mosaic *RUNX1* loss most probably due to the loss of a derivative chromosome 21. One patient has developed acute myeloid leukemia (AML), and another was diagnosed with RUNX1-FPDMM due to thrombocytopenia onset following T-lymphoblastic lymphoma.

**Conclusion:**

RUNX1-FPDMM is a challenging disease due to its associated increased risk for hematologic malignancies, mainly myelodysplastic syndrome (MDS) or AML. Genetic diagnosis in individuals with thrombocytopenia or functional platelet defects of unknown origin is crucial to offer structured surveillance and patient education. Increased risk of bleeding due to qualitative platelet function defects, particularly granule secretion abnormalities, must be considered when managing patients, especially prior to invasive procedures.

## 1 Introduction

Inherited platelet disorders (IPDs) are a diverse group of conditions that affect platelet count and often platelet function. To date, likely pathogenic variants in more than 70 genes have been identified as potential causes of IPDs, including classical diseases such as Glanzmann thrombasthenia and Bernard–Soulier syndrome (1–3). *RUNX1*-related thrombocytopenia, *ETV6*-related thrombocytopenia, and *ANKRD26*-related thrombocytopenia are three known hereditary thrombocytopenias with an established predisposition to hematologic malignancies (4). All present with lifelong mild-to-moderate thrombocytopenia, but their leukemic risks differ: *RUNX1*-related thrombocytopenia carries the highest reported lifetime risk [≈25–50%, mainly myelodysplastic syndrome (MDS) and acute myeloid leukemia (AML)], *ETV6*-related thrombocytopenia is strongly associated with B-cell acute lymphoblastic leukemia (5), and *ANKRD26*-related thrombocytopenia confers a lower yet significant risk of myeloid transformation (6).

*RUNX1* germline variants, which cause familial platelet disorder with a propensity for myeloid malignancy (RUNX1-FPDMM) (OMIM 601399), were first elucidated in 1999 (7). In addition to the associated predisposition to hematologic malignancies, affected individuals often present with thrombocytopenia, functional platelet defects, and/or autoimmunity long before malignancy might develop (8). Due to the functional platelet defects, clinical observation frequently shows a more pronounced bleeding type than the often only mildly reduced platelet counts in RUNX1-FPDMM. RUNX1-FPDMM follows an autosomal dominant mode of inheritance.

Genetic diagnostics has become routine in suspected or diagnosed malignant diseases such as AML or MDS. While primarily searching for disease-associated somatic genetic alterations, further assessment of identified variants can allow the identification of underlying genetic germline alterations such as *RUNX1* variants. In contrast, comprehensive genetic evaluations for mild, unexplained thrombocytopenia and/or pronounced bleeding tendency have only recently become a standard of care in some clinics. This shift reflects a more cautious diagnostic approach, where clinicians are less likely to diagnose idiopathic thrombocytopenic purpura (ITP) without first excluding potential genetic causes.

The involvement of (pediatric) hemostaseologists can uncover not only thrombocytopenia but also associated conditions such as platelet storage pool defects, which may explain bleeding symptoms. In *RUNX1* deficiency, predominantly a *δ*-granule secretion defect and a mild *α*-granule secretion defect have been described, which may contribute to a more noticeable bleeding abnormality (9, 10). Further genetic analysis in such cases may identify pathogenic *RUNX1* variants, pointing to an underlying oncogenetic predisposition. Recognizing the clinical importance of these findings, a RUNX1 registry and variant database was established in 2021 to support the classification of both germline and somatic *RUNX1* alterations (11). Additionally, the National Human Genome Research Institute RUNX1-FPD Natural History Study is actively accruing more longitudinal cases to genotype and phenotype families with RUNX1-FPDMM (12).

*RUNX1* is a transcription factor subunit that plays a pivotal role in regulating hematopoiesis and facilitating megakaryocyte differentiation. *RUNX1* is located at chromosome 21q22.12. Approximately 25%–50% of individuals with RUNX1-FPDMM will develop malignancy (9, 13, 14), mainly MDS and AML and less frequently acute lymphoblastic leukemia (ALL) or lymphoma or other hematologic malignancies. Age of malignancy onset seems highly variable, ranging from 4 to 77 years (median: 36.5 years) in a recently presented retrospective EU cohort (15). Other publications show similar median ages at malignancy onset (16, 17).

Next-generation sequencing (NGS) has been essential for analyzing genetic alterations in multiple genes associated with the heterogeneous IPDs. However, understanding diagnostic challenges and interpreting results are crucial for establishing accurate diagnoses and making informed recommendations for further diagnostics and treatment (8, 18). For example, the cohort of RUNX1-FPDMM comprises a relevant group of families with copy number alterations (CNA); therefore, copy number variant (CNV) analysis is necessary to detect these cases (19).

In this study, we discuss the cases of six families with germline *RUNX1* variants/alterations, some with additional bleeding tendency due to a platelet granule secretion defect. We illustrate the partially different phenotypes and focus on diagnostic pitfalls while analyzing *RUNX1* alterations.

## 2 Materials and methods

### 2.1 Patients

Patients were referred for diagnostic workup for thrombocytopenia (except P5), and clinical data were collected by a retrospective chart review. This study was approved by Albert-Ludwigs-University Freiburg’s institutional review board (EK584/17 and EK222-20). P5 was registered to the European Working Group of Myelodysplastic Syndrome (EWOG-MDS)-2006 study group (EWOG-MDS 2006 # NCT00662090). Informed consent was obtained from patients and parents for germline genetic analysis. All procedures were conducted in accordance with the Declaration of Helsinki.

### 2.2 Laboratory analyses

#### 2.2.1 Platelet count and platelet aggregometry analyses

Platelet count was measured using an automated cell counter (Sysmex KX-21 N, Norderstedt, Germany). Platelet-rich plasma (PRP) and platelet-poor plasma (PPP) were obtained by centrifugation of citrate-anticoagulated blood samples for 10 min at room temperature. Using the APACT 4 (LABiTec, Ahrensburg, Germany), light transmission aggregometry (LTA) was performed after stimulation with collagen (2 μg/mL; Takeda, Linz, Austria), adenosine diphosphate (ADP; 4 μmol/L; Sigma-Aldrich, St. Louis, MO, USA), epinephrine (8 μmol/L; Sanofi-Aventis, Frankfurt, Germany), and ristocetin (1.2 mg/mL; American Biochemical and Pharmaceutical LTD, Frankfurt, Germany). The cutoff for impairment was < 60%, and LTA was only taken into consideration in case of a platelet count > 100 G/L due to impairment of this method in case of more severe thrombocytopenia.

#### 2.2.2 Flow cytometry analyses (FC)

Platelet flow cytometry analyses were performed in Freiburg using FACS Calibur (Becton Dickinson, Heidelberg, Germany) (20). Diluted PRP aliquots (5 × 10^7^ platelets/mL) were fixed and stained with fluorescein isothiocyanate (FITC)-labeled monoclonal surface antibody against CD41 (GPIIb/IIIa-complex), CD42a (GPIb/IX), and CD42b (GPIb) (Coulter, Immunotech, Marseille, France). FITC-labeled anti-VWF (Bio-Rad AbD Serotech, Puchheim, Germany) and Alexa Fluor 488-labeled anti-fibrinogen (Invitrogen, Waltham, MA, USA) were used to stain the platelets. The platelets were also stained with monoclonal FITC-labeled anti-CD62 (P-selectin) and anti-CD63 antibodies (lysosomal membrane-associated glycoprotein 3, LAMP-3; Immunotech, Marseille, France). In the presence of 1.25 mM Gly-Pro-Arg-Pro (Bachem, Bubendorf, Switzerland), diluted PRP (5 × 10^7^ platelets/mL) was stimulated with a number of several concentrations of thrombin (0, 0.05, 0.1, 0.2, 0.5, and 1 U/mL; Siemens Healthineers, Marburg, Germany) to conduct the CD62 and CD63 expression analyses. A healthy day control (shown in blue color in the following FC figures) was tested together with the respective patient, and the results were reported descriptively according to the recommendations of the S2K guideline of platelet function analysis (21). Additionally, we included data of a healthy control pool (6 controls in a total of 20 measurements) shown in gray in the following FC figures. The data are expressed as a mean ± standard error of the mean (SEM).

FC analyses were conducted in Dresden on the siblings from family 4 using a FACS Lyric-Cytometer (Becton Dickinson, Heidelberg, Germany). PRP samples were stimulated with 50 μM ADP (Stago Deutschland GmbH, Düsseldorf, Germany) or 50 μM Thrombin Receptor–Activating Peptide (TRAP) (LOXO GmbH, Dossenheim, Germany), and the stimulation was stopped by the addition of 1 mL of Cellwash (Becton Dickinson, Heidelberg, Germany). The samples were then stained with the following antibodies: PAC1 (FITC), CD62P (PE), CD63 (Alexa Fluor), and CD41a (APC-H7) (all Becton Dickinson, Heidelberg, Germany). Aliquots were incubated with 3.4 μM mepacrine (Merck KGaA, Darmstadt, Germany) for the mepacrine uptake and release prior to the stimulation process with ADP/TRAP.

#### 2.2.3 Molecular genetic analyses

To extract genomic DNA from ethylenediaminetetraacetic acid (EDTA) blood, we applied standard procedures using the QIAamp^®^ DNA Blood Mini Kit (Qiagen GmbH, Hilden, Germany). Panel sequencing (95 genes) was performed using a custom-designed hybridization-based enrichment kit (Illumina, San Diego, USA), followed by sequencing on a MiSeq sequencer (Illumina). Data were analyzed using Sequence Pilot (JSI medical systems, Germany). We used supporting software ALAMUT^®^ (v.2.15), pathogenicity prediction (CADD; Combined Annotation Dependent Depletion), and occurrence in population (gnomAD) and disease databases (HGMD public version, ClinVar) to classify the variants. Variant classification was performed following the American College of Medical Genetics and Genomics/Association for Molecular Pathology (ACMG/AMP) guidelines (22). RUNX1 alterations were classified under consideration of the ClinGen Myeloid Malignancy Expert Panel (MM VCEP) Specifications to the ACMG/AMP Variant Interpretation Guidelines Version 2 (23). For *RUNX1,* we used the main selected transcript variant NM_001754.5, which encodes for isoform c. For P5, genetic analysis was performed in Hannover using a 47-gene custom somatic panel (gene list in Supplementary material), and targeted Sanger sequencing for germline testing from buccal swab and hair follicle was performed at the reference and diagnostic laboratory of the EWOG-MDS/EWOG-SAA study group in Freiburg.

Chromosomal microarray analysis (CMA) was performed for P1 using Infinium^®^ CytoSNP-850 K BeadChip v1.2 (Illumina, San Diego, USA) according to the manufacturer’s instructions at the MGZ Munich. Scanning and image acquisition were performed using an Illumina iScan microarray scanner. Data analysis was performed using BlueFuse Multi software v4.5 (Illumina, San Diego, USA). For copy number analyses of P2.1, P2.3, and P6, high-resolution microarray-based comparative genomic hybridization (aCGH) was performed at Hannover Medical School. In comparison to a reference DNA pool, the samples were analyzed using a custom 400 k oligo eArray (design 84704, Agilent Technologies, Waldbronn, Germany) in accordance with the manufacturer’s instructions. Fluorescence signals were scanned with a dual laser microarray scanner, SureScan Dx, and analyzed with Feature Extraction 12.1.1.1 and CytoGenomics 5.0.0.14 (all Agilent Technologies).

## 3 Results

The results for all investigated patients (*n* = 10) of 6 families are summarized in Table 1.

**Table 1:** Results of laboratory investigations in RUNX1-FPDMM case series.

Family.Case No. (Age)	Platelet count G/L [Median (min−max)]^1^	Light transmission aggregometry^2^			Flow cytometry	*RUNX1*-variants^4^ and cytogenetic alterations	Malignancy	Family history for malignancy
1 (2 y)	131 (109–136)	Collagen	14%	Platelets: 151 G/L	Reduced CD62 and CD63 exposure	arr[GRCh38] 21q22.11q22.12(33,931,656_35,493,436)x1dn; 1.56 Mb deletion including *RUNX1*	No	Yes
		ADP	45%					
		Epinephrine	17%					
2.1 (7 y)	144 (106–175)	Collagen	12%	Platelets: 118 G/L	Reduced CD62 (mildly) and CD63 (more pronounced) exposure	arr[GRCh38] 21q22.12(34,961,707_35,875,169)x1; 913 kb deletion including exons 1 and 2 of *RUNX1* transcript variant 1	No	Yes
		ADP	41%					
		Epinephrine	0%					
2.2 (62 y)	142 (106–150)	Collagen	0%	Platelets: 106 G/L	Reduced CD62 and CD63 exposure		AML	Yes
		ADP	42%					
		Epinephrine	26%					
2.3 (40 y)	104	Collagen	54%	Platelets: 104 G/L	Borderline CD62 exposure and reduced CD63 exposure only compared to day control		No	Yes
		ADP	53%					
		Epinephrine	17%					
3.1 (1 y)	40	n/a			N/A	c.602G > A p.(Arg201Gln)	No	Yes
3.2 (36 y)	60	n/a			N/A		No	Yes
4.1 (10 y)	115	n/a			CD62 exposure not impaired, borderline CD63 exposure^3^	c.637C > T p.(Gln213*)	No	No
4.2 (6 y)	208	n/a			CD62 exposure not impaired, borderline CD63 exposure^3^		No	No
5 (17 y)	127 (70–152)	Collagen	41%	Platelets: 117 G/L	Not impaired	c.460C > T p.(Gln154*)	T-LBL	No
		ADP	28%					
		Epinephrine	7%					
6 (13 y)	120	Collagen	71%	Platelets: 122 G/L	Not impaired	mos 46,XY,r(21)(p11;q22.1)[48]/45,XY,-21[4]. arr[GRCh38] 21p12p11.2(5,017,349_7,747,685)x1,21p11.2q22.13(7,753,652_37,446,031)x1[0.59],21q22.13q22.3(37,446,032_46,675,944)x1, mosaic deletion and monosomy 21 with RUNX1 loss in approximately 59% based on aCGH data; follow-up analyses: PHA-stimulated culture: mos 46,XY,der(21)del(21)(p12)del(21)(q22.1)[8]/45,XY,-21[2]. ish der21(RUNX1+).nuc ish 21q22(RUNX1x1)[24/100],21q22.13q22.2(AFMO16XE5/D21S341/D21S342x1)[87/100]; blood smear: nuc ish 21q22(RUNX1x1)[82/100],21q22.13q22.2(AFMO16XE5/D21S341/D21S342x1)[100/100]	No	No
		ADP	53%					
		Epinephrine	44%					

1 In case only 1–2 measurements available: minimal platelet count, in P5: platelet count only after intensive chemotherapy taken into account.

2 Reduced aggregation: < 60%.

3 After stimulation with ADP.

4 Refseq for given HGVS variant description: NM_001754.5.

PHA, phytohemagglutinin.

### 3.1 Platelet FC analyses

**Figure 1:**
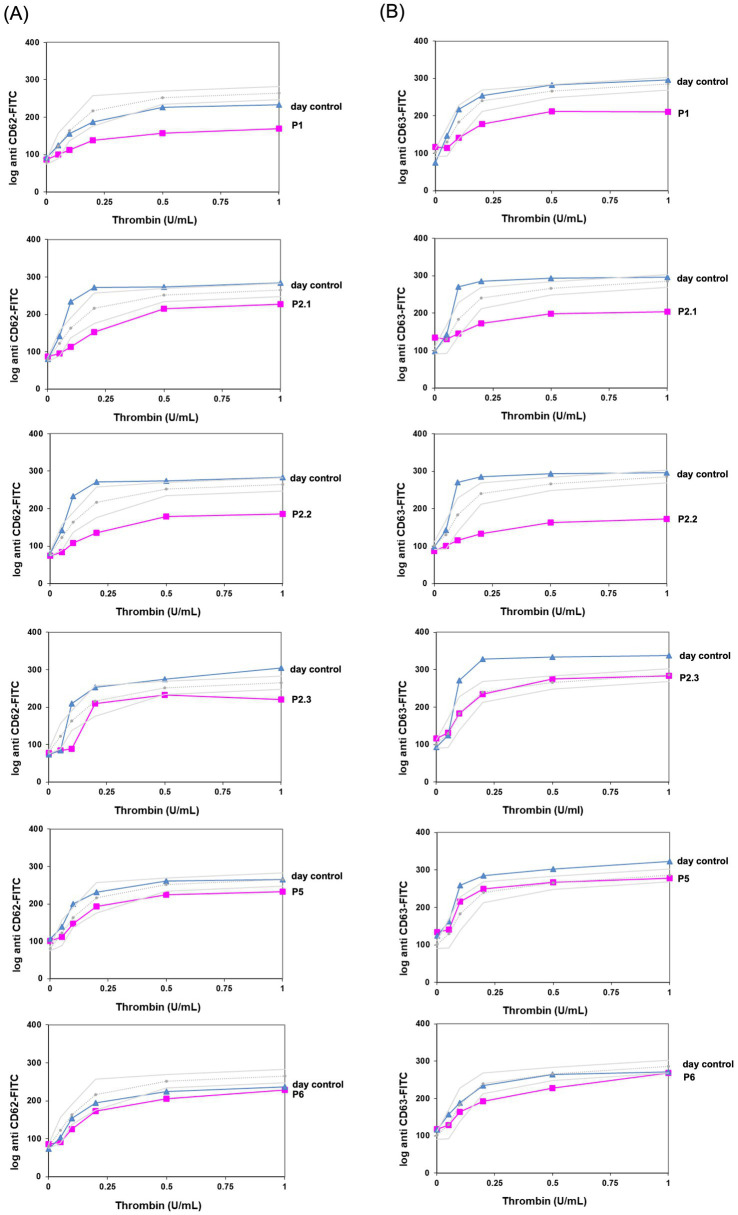
Flow cytometric platelet granule secretion shown for patients with FC analysis in Freiburg. Expression of CD62 **(A)** and CD63 **(B)** compared to healthy controls after stimulation with thrombin (concentrations: 0, 0.05, 0.1, 0.2, 0.5, and 1.0 U/mL). Data of patients and controls [day control and data of 6 controls in 20 independent measurements as mean ± standard error of the mean (SEM)] are expressed as logarithmic arbitrary units (logAU) of stained unstimulated and stimulated platelets.

At the hemostaseology laboratory in Freiburg, we performed platelet FC for six patients of the four families (Figure 1). The two siblings from family 4 were investigated in Dresden (data not shown). In FC analysis, three of the eight investigated individuals showed a clear granule secretion defect (Figure 1: P1, P2.1, and P2.2). Granule secretion defect was not detectable in three individuals (P4.1, P4.2, data not shown; Figure 1: P5), and FC analysis could not be performed for two individuals (family 3).

### 3.2 Patients’ characteristics

#### 3.2.1 Family 1: *RUNX1*-gene deletion

**Figure 2:**
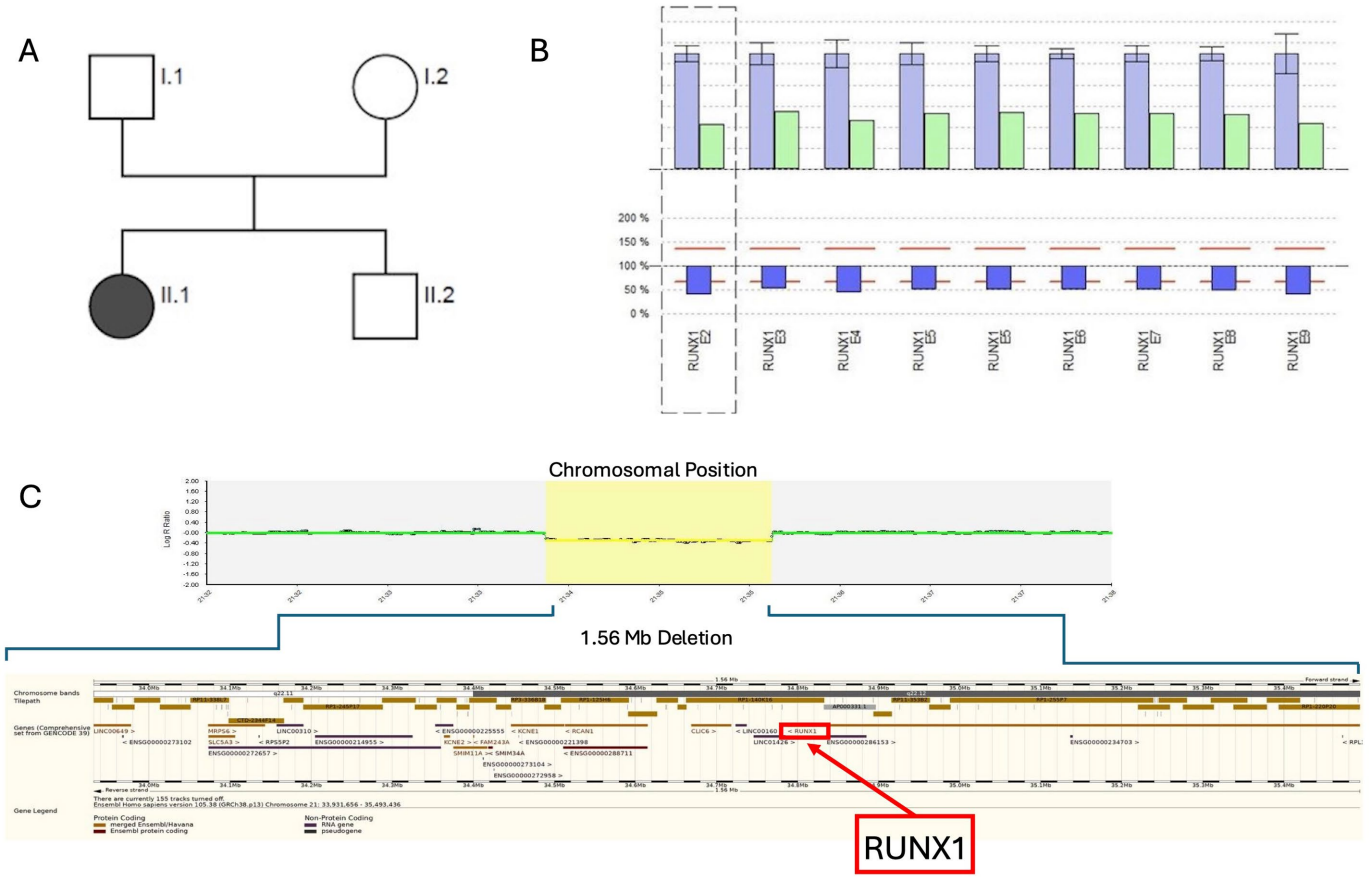
Pedigree and genetic analysis for family 1. **(A)** Genetic pedigree of family 1; black circle II.1 indicates the affected child (P1). **(B)** SeqPilot^®^ CNV analysis: reduced copy number for the RUNX1 exons for the index patient (green columns) compared to the patients analyzed in the same enrichment (blue), indicating a heterozygous *RUNX1*-gene deletion. **(C)** Chromosomal microarray analysis: 1.56 Mb deletion (21q22.11q22.12 shown as a red bar) including *RUNX1*.

We present the case of a 2-year-old girl (P1) who presented to an outpatient coagulation clinic due to recurrent hematomas (International Society on Thrombosis and Hemostasis Bleeding Assessment Tool (ISTH BAT) score 2) (24) (Figure 2A). Her mother reported a family history of leukemia of unknown specificity in siblings of the maternal grandparents. Thrombocytopathy was suspected based on reduced platelet function analysis (PFA-100) results, and the person was referred for further analysis.

At the first presentation at our clinic, a persistent thrombocytopenia (109–136 G/L) and thrombocytopathia (LTA: impaired after stimulation with collagen, ADP, and epinephrine) were detected (Table 1), and the platelet size was normal [mean platelet volume (MPV): 8.8 fl (7–12 fl)]. Platelet FC analyses were performed for P1. After activation with thrombin in different concentrations (0.1–1 U/mL), the platelets showed reduced platelet CD62 (P-selectin, alpha-granule marker, Figure 1A) and CD63 (delta-granule and lysosomal marker) exposure (Figure 1B) compared to healthy controls, indicating a combined platelet granule secretion defect. Then, NGS panel analysis was performed, and the copy number analysis (CNV) tool from the sequencing software SeqPilot^®^ identified a heterozygous *RUNX1*-gene deletion (Figure 2B). To validate the finding and define deletion size, we performed a microarray analysis. This investigation identified a 1.56 Mb deletion (i.e., arr[GRCh38] 21q22.11q22.12(33,931,656_35,493,436)x1dn), which comprises *RUNX1* (Figure 2C, Table 1).

By chromosomal microarray analysis, the deletion was not identified in the parents. Additionally, we investigated the platelets of the index person’s (IP) mother. Her platelet count was within the normal range (288 G/L), and LTA and FC analysis were normal. The recently born brother has a normal platelet count and does not carry the deletion. The family investigations suggest a *de novo* occurrence of the 1.56 Mb deletion in the IP, unless there is a germline mosaic in one of the parents.

A bone marrow aspiration showed no signs of AML or MDS at the time of diagnosis but slightly reduced cellularity of the marrow fragments and very dysplastic megakaryocytes (many very small megakaryocytes with round, separated, or unlobulated nuclei). Microarray analysis performed for bone marrow showed a deletion of 21q22.11q22.12. Regular hematological checkups, including differential blood counts every 3 months and annual bone marrow aspirations, were recommended for the affected individual.

#### 3.2.2 Family 2: microdeletion in *RUNX1*

The IP, a 7-year-old boy (P2.1), was first evaluated at our clinic for persistent thrombocytopenia (106–175 G/L), which was initially detected during routine blood tests for atopic dermatitis. The family reported recurrent hematomas (ISTH BAT score 3), but no additional bleeding manifestations were observed. Thrombocytopenia was also present in his father (P2.2; 106–150 G/L) and 30-year-old half-sister (P2.3; 104 G/L), both of whom remained asymptomatic.

**Figure 3:**
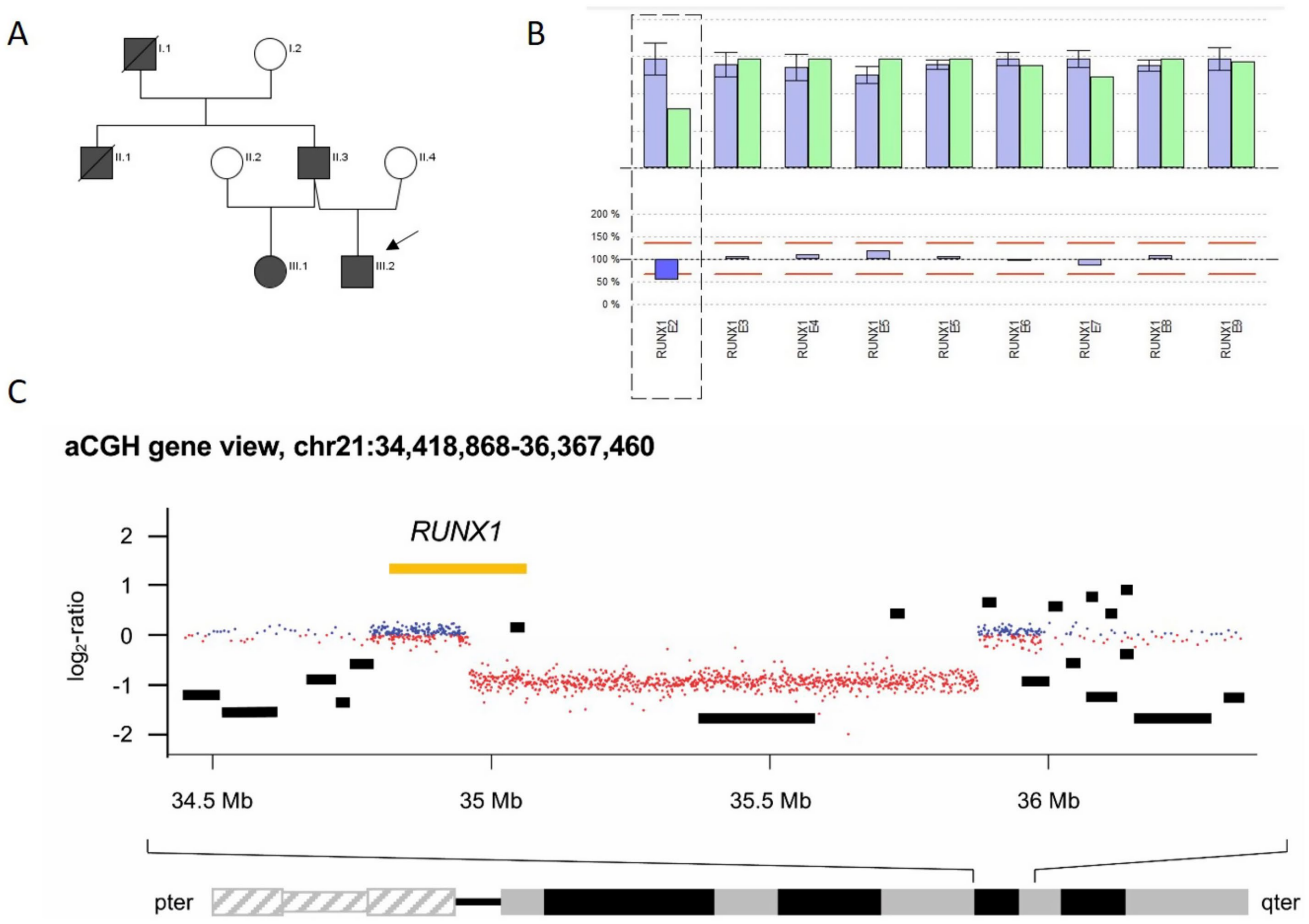
Pedigree and genetic analysis for family 2. **(A)** Pedigree of family 2: Marked in black are family members affected with thrombocytopenia (index patient III.2 marked with an arrow ≙ P2.1) and his half-sister III.1 ≙ P2.3 or hemato-oncologic disease [their father II.3 ≙ P2.2, the deceased uncle (II.1), and grandfather (I.1) of P2.1 and P2.3]. **(B)** CNV analysis in SeqPilot^®^ shown for P2.1: reduced coverage of the first coding exon 2 of *RUNX1* (transcript NM_001754.5) for P2.1 (green columns) compared to individuals analyzed in the same enrichment (blue) and NGS run, indicating a heterozygous deletion of exon 2. The non-coding exon 1 was not covered by the NGS analysis. **(C)** High-resolution aCGH gene view of chromosome 21 from 34,418,868 to 36,367,460 (GRCh38) for P2.3 plotted above the ideogram of chromosome 21, indicating a heterozygous deletion of 913 kb including exons 1 and 2 of *RUNX1* transcript variant 1 encoding RUNX1c on the minus strand.

The paternal family history was remarkable: the grandfather died of MDS/AML with an MLL deletion, and a paternal uncle passed away in 2015 shortly after a third stem cell transplant for MDS/AML (Figure 3A). This transplant had been sourced from his haploidentical brother (P2.2) at a time when the *RUNX1* microdeletion had not yet been recognized.

Before the donation, the father (P2.2) underwent bone marrow evaluation that did not reveal any significant abnormalities (cytomorphology: physiological; histology: normocellular hematopoietic marrow), although he was thrombocytopenic (128 G/L). Nine years later, he presented with frequent hematomas and was diagnosed with MDS with excess blasts (leukocyte count (Lc): 4.2 G/L, blasts: 5–10%, hemoglobin (Hb): 12.7 g/dL, Tc: 40 G/L). Allogeneic hematopoietic stem cell transplantation (HSCT) was indicated; however, prior to transplantation, his disease progressed to AML with cutaneous involvement (9 months after MDS diagnosis: Lc: 0.63 G/L, Hb: 7.9 g/dL, platelets: 6 G/L). A myeloid panel revealed a somatic SRSF2 mutation with a variant allele frequency (VAF) of 45%.

The patient subsequently underwent successful HSCT, with normalization of platelet counts observed post-transplant.

Given the significant family history, we conducted extensive analyses, including LTA, FC, and NGS. LTA showed reduced aggregation after stimulation with collagen, ADP, and epinephrine in the IP, the half-sister, and the father, respectively. Additionally, a mild reduction of CD62-P exposure was seen for all three tested family members after stimulation with thrombin (Figure 1A). CD63 exposure was reduced in P2.1 and P2.2 (P2.3 only compared to day control) (Table 1, Figure 1B). The platelet size was normal in all three patients: P 2.1: MPV 10 fl (7–12 fl), P 2.2: MPV 9.8 fl (7–12 fl), and P 2.3: MPV 9.6 fl (7–12 fl). CNV analysis indicated a heterozygous deletion of the first translated exon, exon 2 of the *RUNX1* gene, in the IP (Figure 3B), the father, and the half-sister (data not shown). The deletion in 21q22.12 was confirmed by high-resolution aCGH in the IP (P2.1) and the half-sister (P2.3) (Figure 3C). The deletion has a genomic size of approximately 913 kb and affects exons 1 and 2 of the *RUNX1* isoform c (NM_001754.5).

#### 3.2.3 Family 3: pathogenic variant *RUNX1*

**Figure 4:**
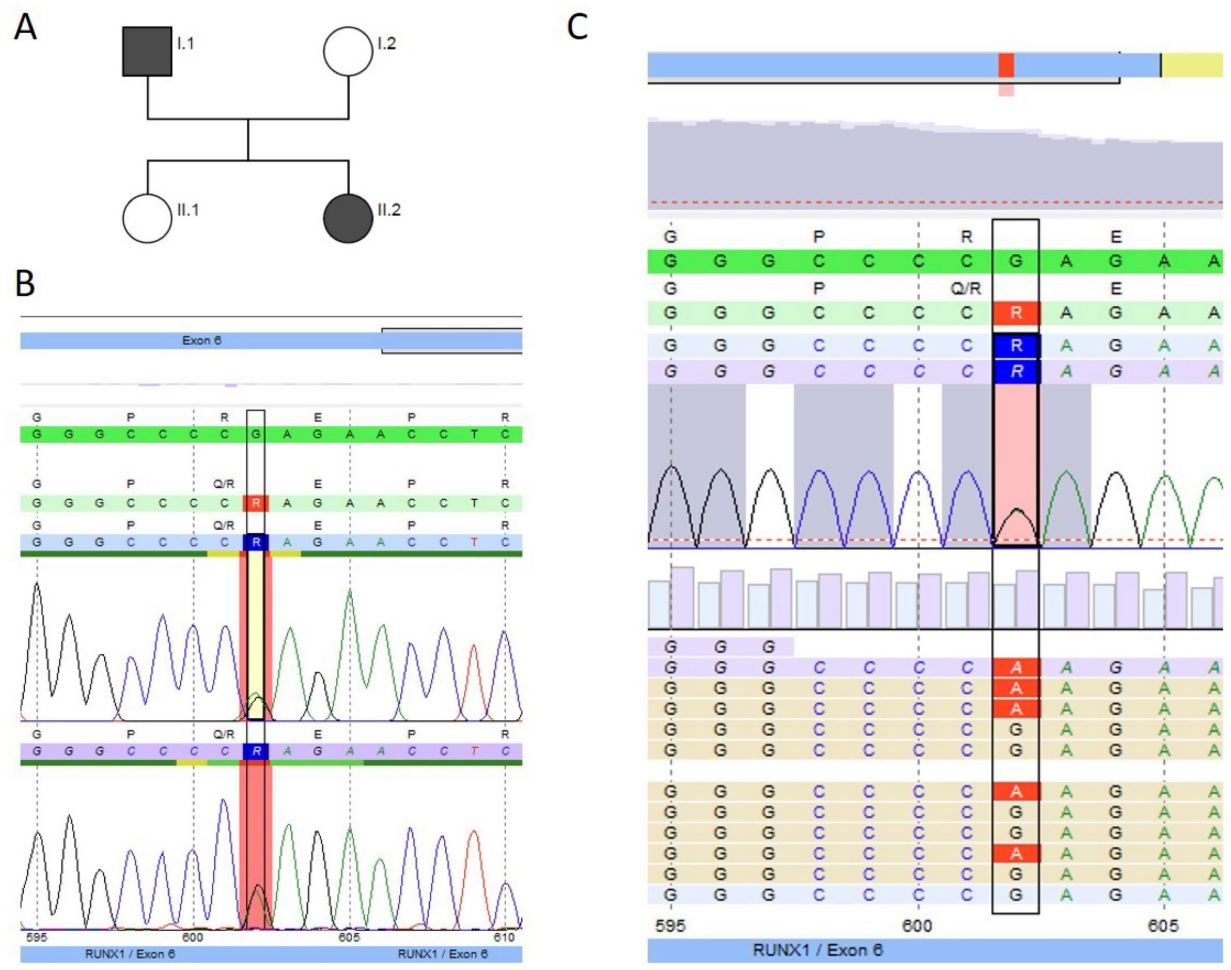
Genetic pedigree and genetic analysis for family 3. **(A)** Pedigree; black indicates the thrombocytopenic father (I.1 ≙ P3.2) and index patient (II.2 ≙ P3.1), both heterozygous carriers of the pathogenic *RUNX1* variant c.602G > A (p. Arg201Gln). NGS panel analysis was performed for the index patient and her parents as trio-analysis, and genotyping was performed using Sanger sequencing for the unaffected sibling II.1, in whom the variant was not detected. **(B)** Sanger sequencing (forward and reverse chromatogram) for the index patient confirmed the pathogenic *RUNX1* variant identified with panel sequencing. **(C)** NGS data for the father showing the c.602G > A pathogenic variant (coverage 49% (440) [51% (198) / 49% (242)]) (software SeqPilot^®^).

The IP (P3.1) was a 1-year-old girl who first presented at an outpatient clinic with neonatal thrombocytopenia and a history of multiple platelet transfusions. Bleeding history revealed petechiae in the neonatal period (ISTH BAT score 2). She did not respond to immunoglobulin; however, her platelet count stabilized over time at approximately 40 G/L, and MPV was normal [10.1 fl (7–12 fl)]. The girl’s father also had thrombocytopenia (60–100 G/L), which had been known since he was 11 years old. He reported petechiae, epistaxis, and bleeding after minor wounds (ISTH BAT score 3). He was misdiagnosed with ITP in his home country. According to the information provided by the patient, he received treatment for the suspected ITP; however, there is no information about his treatment (no medical records) (Figure 4A). The paternal grandmother had lifelong thrombocytopenia and was diagnosed with AML at the age of 67 years and recently died. The father’s sister died at the age of 3 years due to acute lymphoblastic leukemia (ALL); it is unclear whether it was T-cell lymphoblastic leukemia (T-ALL) or B-cell lymphoblastic leukemia (B-ALL).

We performed NGS panel trio-analysis for the IP and her parents, the affected father and the unaffected mother. No material was available from the paternal relatives to perform genetic analysis. A heterozygous pathogenic variant was detected in *RUNX1* (NM_001754.5) exon 6 in the IP and her father (Figures 4B,C) and was confirmed with Sanger sequencing. The substitution c.602G > A (rs74315450) leads to an amino acid exchange at position 201 from arginine to glutamine (p. Arg201Gln). This variant is not present in population databases (gnomAD v2.1.1). The variant has been classified as pathogenic by the ClinGen Myeloid Malignancy Variant Curation Expert Panel (14) and is also listed as pathogenic in ClinVar (Accession: VCV000014464.6). Additionally, the alteration c.602G > A (rs74315450) has been described in individuals with a personal and/or family history of platelet dysfunction, mild to moderate thrombocytopenia, and myeloid malignancies. It has also been observed that segregation with disease occurs in related individuals (9, 25). There was no platelet function analysis performed in family 3. The healthy sibling did not carry the *RUNX1* variant.

#### 3.2.4 Family 4: non-sense variant *RUNX1*

Two siblings (10-year-old brother P4.1 and his 6-year-old sister P4.2) were referred for genetic analysis due to mild thrombocytopenia. P4.1 presented with platelet counts between 44 and 168 G/L, and his sister, P4.2, presented with platelet counts between 85 and 242 G/L. P4.1 had minimally reduced platelet size, with an MPV of 8.3 (9–13 fl), and P4.2 had normal platelet size, with an MPV of 9.1 (9–13 fl). The brother (P4.1) had postnatal hematoma at the legs and body trunk and had received a platelet transfusion (ISTH-BAT score 1). Additionally, the boy had bilateral postaxial polydactyly and an ulnar skin appendage on the left hand. The sister (P4.2) had postnatal hyposphagma on both sides but no intracranial hemorrhage (ISTH BAT score 1) and postaxial polydactyly on the left foot. There was no genetic testing for the postaxial (bilateral) polydactyly performed in both children. The paternal grandmother had formerly received the diagnosis of nonspecific thrombocytopenia and was never genetically tested.

**Figure 5:**
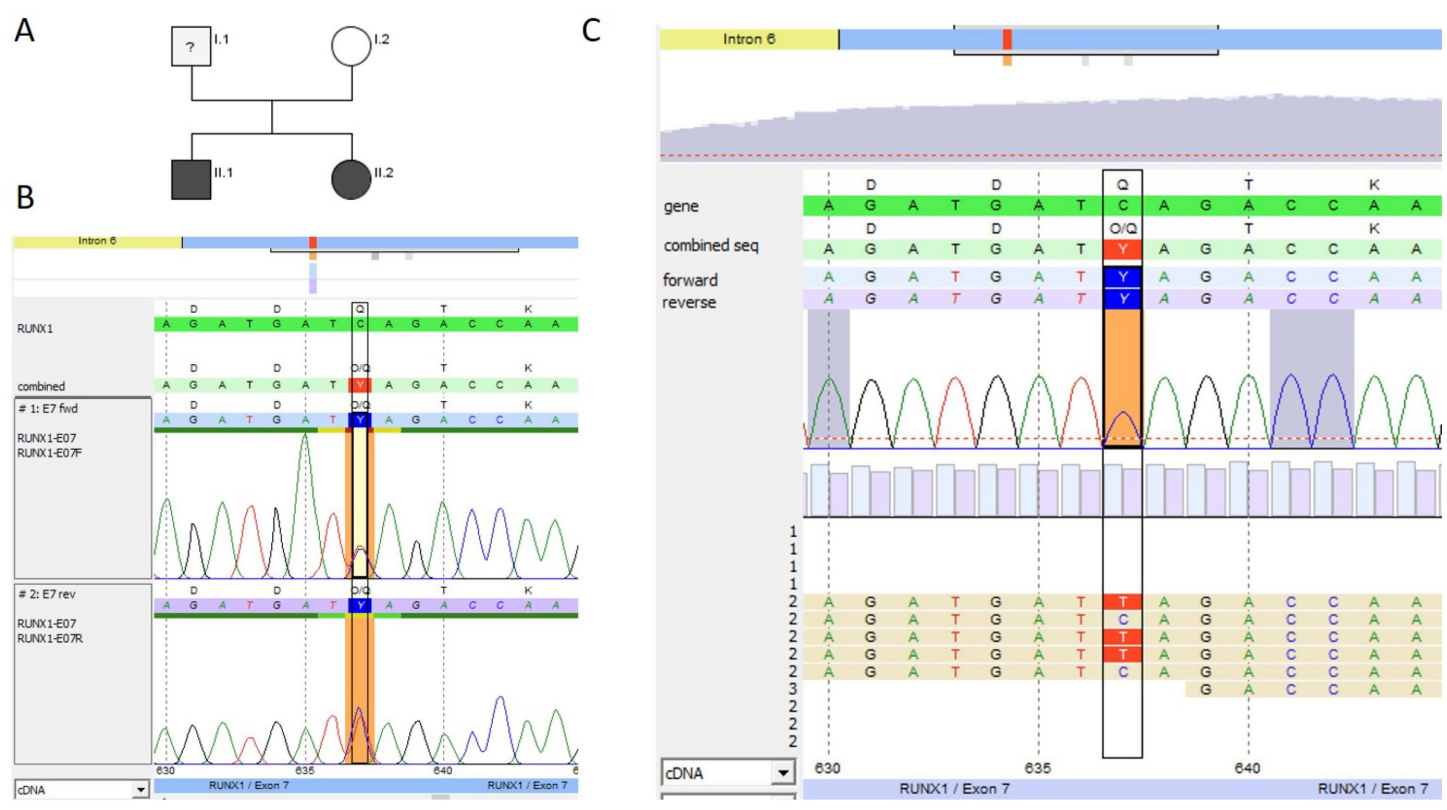
Genetic pedigree and genetic analysis for family 4. **(A)** Pedigree; black indicates the siblings (II.1 ≙ P4.1 and II.2 ≙ P4.2), both heterozygous carriers of the *novel* pathogenic *RUNX1* (NM_001754.5) non-sense variant c.637C > T p.(Gln213*). NGS panel analysis was performed for both siblings. **(B)** Sanger sequencing (forward and reverse chromatogram) for the brother P4.1 confirmed the non-sense mutation in *RUNX1* identified with panel sequencing. **(C)** NGS data for the sister P4.2 showing the c.637C > T pathogenic variant (coverage 50% (343) [50% (180) / 50% (163)]) (software SeqPilot^®^).

We performed NGS panel analysis and identified a heterozygous non-sense variant in both affected siblings, confirmed with Sanger sequencing (Figure 5). The nucleotide exchange c.637C > T in exon 7 leads to a premature STOP codon at position 213. The non-sense variant is not listed in the population database gnomAD (v2.1.1), nor in dbSNP or in ClinVar (accessed 6/27/2024), and, therefore, is considered *novel*. According to the ACMG/MM VCEP criteria, we classified the variant as pathogenic (PVS1, PS4_supporting, PM5_supporting, PM2_supporting; 11 points).

After identification of the *RUNX1* non-sense variant, the siblings were transferred to the hematologic outpatient clinic of the university hospital in Dresden for family counseling and platelet FC (e.g., CD62-P and CD63 exposure after stimulation with TRAP or ADP). Both siblings showed borderline normal CD63 exposure after stimulation with ADP and normal CD62-P exposure (data not shown).

Sanger sequencing for the *RUNX1* non-sense variant showed that the mother was not a carrier of this variant. The father did not want to get tested, and no blood counts are available so far. He was not investigated, but surveillance was offered.

#### 3.2.5 Family 5: non-sense variant *RUNX1*

The patient (P5) was diagnosed with stage III T-lymphoblastic lymphoma (T-LBL) at the age of 11 years. The blood count at the time of diagnosis showed mild thrombocytopenia (127 G/L) with normal hemoglobin and leukocyte counts and normal platelet size, with an MPV of 9.8 fl (7–12 fl). Treatment included polychemotherapy according to the NHL-BFM 2012 protocol. The personal medical history revealed frequent nosebleeds at the age of 8–10 years (ISTH BAT score 1), but no blood count was available before diagnosis of the T-LBL. Chemotherapy was well-tolerated with transfusion dependency for platelets in the expected range and full hematological recovery between chemotherapy blocks.

**Figure 6:**
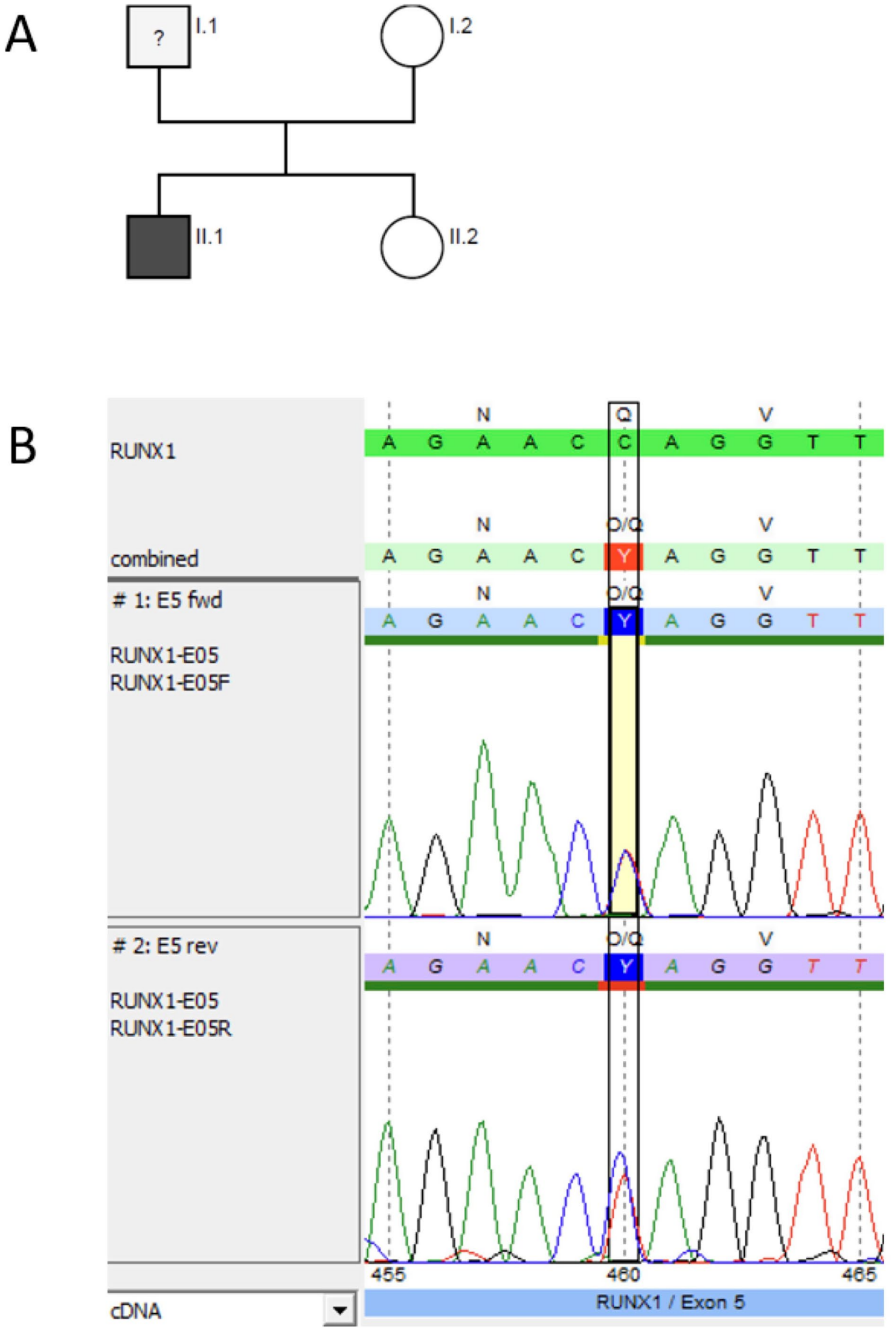
Pedigree and genetic analysis for P5. **(A)** Genetic pedigree for family 5. **(B)** Germline testing (DNA from hair follicles) using Sanger sequencing (forward and reverse chromatogram) for P 5 confirmed the pathogenic *RUNX1* (NM_001754.5) non-sense variant c.460C > T p.(Gln154*) identified via NGS as a germline variant (software SeqPilot^®^).

During routine follow-up, mild persistent thrombocytopenia (100–150 G/L) was noted after completed therapy, prompting further diagnostic evaluation, including a bone marrow biopsy. There were no signs of secondary MDS but reduced non-dysplastic megakaryocytes in the bone marrow. Somatic genetic testing of bone marrow revealed the heterozygous *RUNX1* non-sense mutation NM_001754.5:c.460C > T p.(Gln154*), VAF: 48%, and germline origin was confirmed by Sanger sequencing in DNA of hair follicles (Figure 6B). The non-sense variant is known to dbSNP (rs2146361411) but not listed in the population database gnomAD (v2.1.1) and ClinVar (accessed 2/17/2025). According to the ACMG/MM VCEP criteria, we classified the variant as pathogenic (PVS1, PS4_supporting, PM5_supporting, PM2_supporting; 11 points).

Platelet analyses revealed reduced function in LTA (collagen, ADP, and epinephrine) but no reduction of granule secretion compared to healthy controls in FC (Figure 1).

The patient’s lymphoma remains in remission, and he has no significant symptoms.

After genetic counseling, further genetic tests were arranged for family members, revealing that the mother and sister are not carriers of the *RUNX1* non-sense variant. The father does not want to get tested, and no clinical information is available (Figure 6A). After intensive discussions, the patient opted for regular surveillance, including full blood counts every 3–6 months and an annual bone marrow investigation.

#### 3.2.6 Family 6: ring chromosome 21 r(21)

The individual (P6) was referred due to a known ring chromosome 21 diagnosed as a mosaic: mos 46, XY,r (21)(p11;q22.1)[48]/45, XY,-21[4], FISH: 1 × 45, XY,-21; 3×46, XY,r(21)(p11;q22.1) (from cultured peripheral venous blood). Further genetic testing had not been performed at the mother’s request at that time point. Because of a known association between r(21) and thrombocytopenia, we were asked to further investigate the cause of the patient’s low platelet counts (120 G/L). The mother reported that the patient experienced easy bruising, epistaxis, and prolonged bleeding after traumatic *skin* cuts, although no bleeding occurred during tooth extraction or hernia surgery (ISTH BAT score 3). In addition to thrombocytopenia, the patient also suffered from treatment-refractory epilepsy, intellectual disability, and sleep disturbance.

Functional analysis showed reduced platelet aggregation in response to ADP and epinephrine in LTA (Table 1), and FC indicated only a minimal reduction in CD62 and CD63 exposure after stimulation with thrombin (Figure 1). The platelet size was normal, with an MPV of 11.6 fl (7–12 fl).

**Figure 7:**
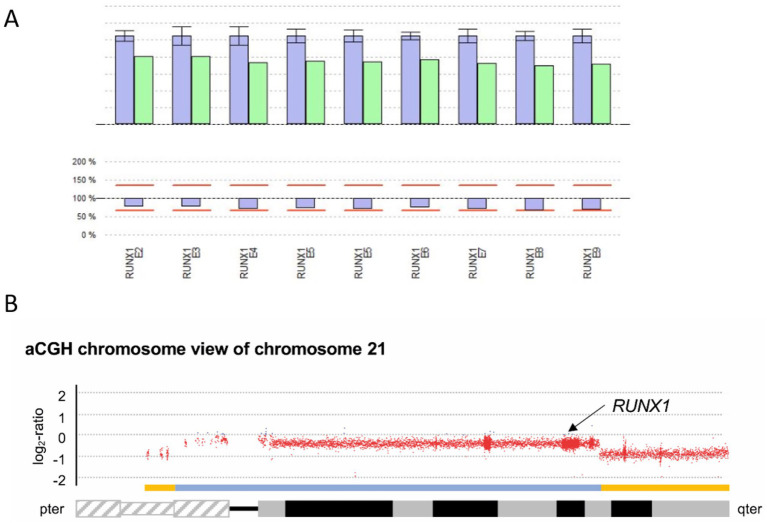
Genetic analysis for P6. **(A)** CNV analysis SeqPilot^®^: Reduced *RUNX1*-gene coverage for P6 (green columns) compared to individuals analyzed in the same enrichment (blue) and NGS run. **(B)** aCGH chromosome view of chromosome 21 displaying the heterozygous deletions in 21p and 21q highlighted by the yellow bars above the ideogram of chromosome 21. The deletion affecting the *RUNX1* locus (black arrow) is indicated by the blue bar.

The imbalance of the *RUNX1* gene, which is located on chromosome 21, is displayed in the NGS data CNV analysis (SeqPilot^®^). The program does not call the reduction a deletion; however, the analysis shows a lower coverage of all *RUNX1* exons (Figure 7A), which is probably due to mosaicism of the ring chromosome.

For further characterization of the previously identified mosaic in this patient, consisting of one cell population with a loss of chromosome 21 and another with a previously reported ring chromosome r(21), an aCGH was performed using DNA extracted from non-cultured peripheral blood to more precisely determine the copy number variations present (Figure 7B).

**Table:** 

arr[GRCh38] of P6: 21p12p11.2(5,017,349_7,747,685)x1,21p11.2q22.13(7,753,652_37,446,031)x1[0.59],21q22.13q22.3(37,446,032-46,675,944)x1

In the aCGH, regarding the losses of genetic material from chromosome 21, a signal constellation was observed that would be consistent with two mosaic cell populations, as previously shown by cytogenetic studies. On the one hand, a heterozygous-appearing loss of genetic material from the short arm of chromosome 21 distal to the breakpoint at 21p11.2 with a genomic size of approximately 2.7 Mb was detected, as well as a heterozygous-appearing loss of genetic material from the long arm of chromosome 21 distal to the breakpoint at 21q22.13 with a genomic size of approximately 9.2 Mb. Additionally, a loss of genetic material proximal to the breakpoints in 21p and 21q with a genomic size of approximately 29.7 Mb was detected. Based on the mean log_2_ ratio for this region, and assuming a heterozygous loss, this would correspond to a cell fraction of 59% carrying this loss. The *RUNX1* locus is located proximal to the breakpoint detected in 21q22.13, while the locus-specific probe LSI 21 used in previous FISH analysis is located distal to this breakpoint. We assume that the distal losses in 21p and 21q reflect the ring chromosome detected in the cytogenetic analysis and that the less prevalent deletion corresponds to the described clone with monosomy 21.

The proportions of copy number variations detected by aCGH are not consistent with the results of the cytogenetic analysis from cultured cells, wherein a numerical evaluation, 48 of 52 metaphases (92%) showed r(21), and only 4 of 52 metaphases (8%) showed monosomy 21. Subsequent investigations of peripheral blood comparing FISH on PHA-stimulated cells and blood smears indicated a loss of *RUNX1* in 24 and 82%, respectively (Table 1). Metaphase FISH using a specific RUNX1 probe confirmed that the initially reported r(21) is *RUNX1*-positive and finally appeared more as a derivative chromosome der(21) with terminal deletions in the short and long arms than an r(21).

## 4 Discussion

RUNX1-FPDMM is a hereditary disorder that increases the risk of developing hematologic malignancies, particularly MDS and AML. It often initially presents with thrombocytopenia or platelet dysfunction, which may occur years before any malignancy develops. Patients with unexplained bleeding symptoms are frequently evaluated by hemostaseologists, who play a crucial role in identifying underlying platelet disorders and, increasingly, genetic etiologies. Genetic testing has become an essential component of the diagnostic workup for thrombocytopenia and platelet function disorders and can reveal conditions such as RUNX1-FPDMM, which have significant implications for clinical management beyond thrombocytopenia and/or bleeding tendency. Early recognition of disease progression in affected individuals enables timely diagnosis, more effective counseling, and personalized treatment strategies.

### 4.1 Platelet function analysis in RUNX1-FPDMM

In this case series, investigations were typically initiated due to the presence of thrombocytopenia—with or without bleeding symptoms—and were often accompanied by a positive family history. Platelet counts ranged from as low as 40 G/L to low-normal levels of 208 G/L, which is consistent with previously published data (8, 12). The most frequently reported bleeding symptom was easy bruising. The increased bleeding tendency observed in individuals carrying *RUNX1* variants—often more pronounced than in other forms of mild thrombocytopenia—can be attributed to impaired platelet function, most clearly demonstrated by LTA. In all six patients, LTA revealed impaired aggregation responses to collagen, ADP, and epinephrine. FC analysis identified a distinct granule secretion defect in three of the eight investigated individuals (P1, P2.1, and P2.2). Individuals with whole-gene or exon deletions appeared to exhibit more pronounced granule secretion defects (Table 1), whereas such defects were not detectable in the three individuals harboring non-sense mutations (P4.1, P4.2, and P5).

To date, delta-granule secretion defects have been most commonly reported in RUNX1-FPDMM, although two studies have also described alpha-granule secretion defects (10, 12). In the present case series, reduced CD63 exposure in three of the eight individuals indicated a delta-granule secretion defect; in addition, reduced CD62 exposure in the same individuals (P1, P2.1, and P2.2) suggested a concurrent alpha-granule secretion defect. These findings support the presence of combined granule secretion defects in individuals with *RUNX1* alterations. Notably, in five of the eight investigated individuals, no clear granule secretion defect could be identified by FC (P2.3, P4.1, P4.2, P5, and P6). This observation aligns with the findings of Cunningham et al., who reported reduced dense granules by platelet electron microscopy in 16 of 35 individuals with FPDMM. In their cohort, 27 of 39 (69%) patients exhibited alpha-granule abnormalities, while LTA was impaired in all 18 individuals tested, including two with normal platelet counts (12). To date, no definitive genotype–phenotype correlations have been established, although larger studies and longitudinal follow-up may help to clarify these associations. Practically, a comprehensive bleeding history and functional platelet analysis should be obtained in individuals with RUNX1-FPDMM, particularly prior to surgical procedures.

### 4.2 Diagnostic challenges

In all but one patient (P4.1), thrombocytopenia was observed with normal platelet size. This finding represents a characteristic feature shared by the three major thrombocytopenia-related disorders associated with an increased risk of hematologic malignancy—*RUNX1*-, *ETV6*-, and *ANKRD26*-related thrombocytopenia. Therefore, in cases of suspected hereditary thrombocytopenia with normal platelet size, one of these three disorders should be considered (4). The presence of additional platelet dysfunction further strengthens the suspicion of RUNX1-FPDMM.

Regarding the molecular diagnostic challenges in RUNX1-FPDMM, conventional sequence analysis alone detects only approximately 80% of pathogenic variants. To identify exon or whole-gene deletions—so-called CNAs—a gene-targeted deletion/duplication analysis is required (8).

This limitation was evident in our small cohort: heterozygous *RUNX1* deletions (P1) and *RUNX1* exon deletions (P2.1, P2.2, and P2.3) were identified as the underlying defects in two pedigrees using CNV analysis and subsequently confirmed by microarray analysis. If these techniques are not performed—as has been recommended for several years—diagnoses of larger *RUNX1* deletions continue to be missed (19).

In pedigree 2, P2.1 and P2.2 had previously been analyzed for *RUNX1* alterations using both amplicon-based and hybridization-based NGS panels; however, CNV analysis was not performed at that time despite a highly suggestive family history. The microdeletion of the first coding exon (exon 2) was initially detected in P2.3 and subsequently confirmed in the other thrombocytopenic family members, P2.1 (IP of the present study) and P2.2 (his father).

P6 represented a particularly interesting case, previously reported to harbor a ring chromosome 21 in mosaicism with a monosomy 21 clone. A comparable case involving monosomy 21 and ring chromosome 21 has been described in the literature, with reported population proportions of 40 and 60%, respectively (26).

In summary, the analysis confirms that the loss of genetic material from chromosome 21 constitutes a disease-causing alteration that adequately explains the phenotype observed in this patient.

Microdeletions in the 21q21–q22 region were identified in 2016 as the cause of the phenotype originally described in 1994 as Braddock–Carey syndrome (MIM 619980), clinically characterized by Pierre Robin sequence, persistent congenital thrombocytopenia, agenesis of the corpus callosum, distinctive facial features, short stature, and severe developmental delay (27).

Given the observed discrepancy between population proportions determined by cytogenetic analysis of cultured cells and by aCGH/NGS on DNA from peripheral blood, additional molecular cytogenetic analyses of blood smears and cultured cells were initiated and confirmed the proliferation advantage of *RUNX1*-proficient cells, indicating, in line with aCGH/NGS data, a heterozygous *RUNX1* loss in a significant proportion of the cells. When possible, non-hematopoietic cells such as cultured skin-derived fibroblasts, as well as bone marrow aspirates or biopsies, will also be examined. So far, the mother of the patient does not agree to take additional tests. Based on findings from the previously reported patient and the current proband, it can be assumed that mosaic loss of *RUNX1* may be sufficient to cause thrombocytopenia but only mild functional platelet defects (P6: slightly decreased LTA, no storage pool defect). Whether mosaic heterozygous *RUNX1* loss is associated with a reduced risk of hematologic malignancy—given that fewer cells carry the pathogenic alteration due to monosomy 21—remains unknown.

Additionally, one missense variant (P3.1 and P3.2) and two non-sense variants (P4.1/P4.2 and P5) were identified in our cohort. The two non-sense variants (pedigrees 4 and 5) have not been previously reported as germline alterations. Due to the premature termination of the protein at codons 154 and 213, these variants are classified as pathogenic according to the MM VCEP criteria (23).

This case series underscores the importance of comprehensive genetic testing strategies—including CNV analysis and aCGH—for the accurate diagnosis of *RUNX1*-related disorders. Furthermore, all patients carrying a *RUNX1* variant should undergo comprehensive platelet function testing (including LTA and FC) at least prior to major surgical interventions.

A key diagnostic challenge in inherited thrombocytopenia lies in their frequent misclassification as acquired disorders, particularly ITP. A thorough medical history—focusing on age of onset, lifelong platelet count stability, bleeding history, and the presence of affected relatives—provides essential clues suggesting a genetic etiology and guiding further diagnostic evaluation (28).

### 4.3 Predisposition and development of malignancy

To date, more than 250 individuals with germline pathogenic variants in *RUNX1* have been reported in the literature (11). Studies by Brown et al., Simon et al., and a recent retrospective European cohort indicate that the lifetime risk of developing a hematologic malignancy ranges from 25% to 50% (13, 15, 29, 30). A minority of affected individuals show no clinical or laboratory abnormalities, suggesting that a number of cases remain undiagnosed (11). Overall, RUNX1-FPDMM is likely underrecognized (13, 30). Earlier studies proposed that certain missense or non-sense variants might act in a dominant-negative manner by impairing DNA binding or transactivation, thereby increasing the risk of hematologic disease (31). However, larger cohort studies have not confirmed this hypothesis, and genotype–phenotype correlations remain under investigation. Consequently, it is not currently justified to communicate differing risk levels based solely on variant type (12, 13). Importantly, heterozygous pathogenic *RUNX1* variants alone are insufficient to cause malignancy; secondary somatic events affecting *RUNX1* or other hematologic malignancy-associated genes are required for progression to MDS, AML, or, more rarely, lymphoid malignancies (16).

The increasing use of NGS-based gene panels in the evaluation of thrombocytopenia will likely result in a growing number of RUNX1-FPDMM diagnoses and may, over time, improve estimates of lifetime malignancy risk—provided that unified surveillance protocols and consistent database documentation continue to be implemented.

A 2021 publication by Homan et al. from the *RUNX1* database reported 56 individuals with a pre-leukemic state (11). The mean age at diagnosis of an *RUNX1* variant was slightly younger (35–40 years) than in the cohort with manifest malignancy (> 40 years). Our study contributes ten additional individuals (including eight children) from six families to this dataset. To date, two patients in our cohort have developed hematologic malignancies—AML in P2.2 and T-LBL in P5. Several other family members not included in this investigation were also affected, predominantly with AML, but also with MDS and other unspecified leukemias. Bone marrow aspirations in our cohort revealed reduced cellularity in both evaluated patients (P1 and P5) and dysplastic megakaryopoiesis in one (P1). Consistent with these findings, the natural history study reported dysmegakaryopoiesis in 42 of 55 patients and reduced cellularity in 17 of 21 pediatric cases (12).

We further describe one patient (P6) with mosaicism involving a rare ring chromosome 21 (or derivative chromosome 21) and monosomy 21, showing slightly reduced *RUNX1* gene coverage in CNV analysis. The deletion, detected in approximately 59% of cells by aCGH, encompasses the *RUNX1* locus and, together with other identified copy-number variations, corresponds to an FPDMM genotype. Additionally, previous reports have described a predisposition to iAMP21-associated ALL (intrachromosomal amplification of chromosome 21 in acute lymphoblastic leukemia) in individuals with r(21) (32).

The expansion of genetic testing from oncology patients to individuals with only mild hematologic abnormalities, such as isolated thrombocytopenia, raises important ethical questions regarding how to communicate genetic predisposition—particularly given the uncertainty about which individuals will ultimately develop malignancy. This uncertainty underscores the need for thorough pre-test education and well-structured post-test surveillance strategies for affected individuals. Comprehensive counseling of patients—and, where applicable, their legal guardians—is essential and legally mandated to ensure informed consent (33). This process also supports patients in understanding their “right not to know.” Accordingly, the establishment of expert consensus guidelines for the management of FPDMM remains a critical need (34). The recent National Society of Genetic Counselors practice resource on counseling for hereditary hematologic malignancy syndromes provides evidence-based recommendations regarding genetic testing, family communication, and surveillance planning (35). It specifically recommends referral to hematology-focused genetic counselors, who possess specialized expertise in managing the complexities of hereditary hematologic malignancy syndromes.

This sensitivity was particularly required in family 4, which had no history of malignant disease. Genetic testing of the children was initiated solely to investigate mild thrombocytopenia. Communicating a diagnosis of a likely pathogenic *RUNX1* variant in such a context poses significant challenges for both clinicians and family members. The father has declined testing thus far, whereas the mother tested negative; therefore, it is likely that the variant was inherited from the father. This result requires careful communication, and affected family members should be closely connected to a hemato-oncological center experienced in managing genetic predisposition syndromes.

The use of thrombopoietin receptor agonists (TPO-RAs) in individuals with germline RUNX1 variants is strongly discouraged, as these agents stimulate megakaryopoiesis and hematopoietic stem cell proliferation, potentially facilitating clonal expansion or leukemic transformation in a genetically predisposed marrow. Their use should therefore be approached with extreme caution or avoided whenever possible (36).

Finally, effective treatment options exist for patients who progress to malignancy. One patient from this cohort and another from our center (not included in this study) successfully underwent HSCT following the development of MDS/AML. Recent data suggest that patients with FPDMM-associated AML may achieve prolonged survival with standard AML therapy, including allogeneic HSCT, compared with those harboring sporadic *RUNX1*-mutated AML (15).

### 4.4 Management

- To date, no official consensus guidelines have been established for the clinical management of RUNX1-FPDMM. However, several existing guidelines provide recommendations on diagnostics and therapy (8, 37–39). Individuals with RUNX1-FPDMM require thorough pre-test counseling and standardized post-diagnostic follow-up procedures, including regular complete blood counts and bone marrow examinations in the event of any abnormalities—either as a baseline assessment or at (bi)annual intervals.
- Due to the underlying functional platelet defect, bleeding tendencies are often more pronounced than would be expected from the typically mild thrombocytopenia. For surgical procedures—particularly those involving mucosal surfaces—a consultation with a specialist in hemostasis is strongly recommended. In the majority of prophylactic settings, antifibrinolytic agents or desmopressin (DDAVP) are sufficient. However, in cases of significant acute bleeding, platelet transfusion may become necessary (40). Because of the potential risk of alloantibody formation, platelet transfusions should be reserved as a secondary treatment option.
- HSCT should be considered for individuals who develop malignant disease. The therapeutic approach and evaluation process may follow standard protocols used in sporadic cases, with the critical exception of donor selection—as emphasized by previous reports and by family 2 in the present case series (39).

## 5 Conclusion

The increasing use of genetic testing in individuals with mild thrombocytopenia is expected to reveal a growing number of cases with germline RUNX1-FPDMM. Our investigation of this cohort supports previous findings that, in addition to thrombocytopenia, all affected individuals also exhibit platelet function defects. These observations have important clinical implications, particularly for surgical interventions or trauma involving mucosal surfaces, where the bleeding risk is often underestimated. Furthermore, this study emphasizes the clinical vulnerability of individuals with germline RUNX1-FPDMM—as well as those with other thrombocytopenia-associated predispositions such as *ETV6* or *ANKRD26*—and underscores the need for dedicated healthcare infrastructure. This should include comprehensive patient education, standardized post-diagnostic counseling, long-term clinical monitoring, and systematic data documentation in disease-specific registries to ensure appropriate management, continuous support, and the development of future evidence-based, personalized care strategies.

## Data Availability

The original contributions presented in the study are included in the article/Supplementary material, further inquiries can be directed to the corresponding author/s.
