# Supplementary material for: RUNX1-FPDMM in families with mild thrombocytopenia and platelet function anomalies: a case series
Source: Front Med (Lausanne). 2025 Dec 11;12:1657054. doi: 10.3389/fmed.2025.1657054 (PMC12739554; doi:10.3389/fmed.2025.1657054)
Supplement: Supplementary file 1 [file Table_1.DOCX]

Gene panel list Freiburg (95 genes)

ACTN1, ADAMTS13, ANKRD26 (including 5´UTR region), ANO6, AP3B1, AP3D1, BLOC1S3, BLOC1S5 (Muted), BLOC1S6 (PLDN, Pallidin), CD36, CD63, COL3A1, CYCS, DIAPH1, DTNBP1, ETV6, F8, FERMT3 (KINDLIN3), FLI1, FLNA, GATA1, GFI1B, GNAS, GNE, GP1BA, GP1BB, GP5, GP6, GP9, HOXA11, HPS1, HPS3, HPS4, HPS5, HPS6, HRG, ITGA2, ITGA2B, ITGB3, JAK2, LYST, MASTL, MECOM, MLPH, MPL, MYB, MYH9, MYO5A, NBEA, NBEAL2, NFE2, ORAI1, P2RY12, PEAR1, PLA2G4A, PLAT, PLAU, PLG, PRF1, PRKACG, PROC, PROS1, RAB27A, RASGRP2, RBM8A, RUNX1, SEPT1-SEPT12, SEPT14, SLFN14, SNAPIN/BLOC1S7, STAT3, STIM1, STX11, STXBP2, TBXA2R, TBXAS1, THPO, TUBB1, UNC13D, VIPAS39, VPS33B, VWF, WAS, WIPF1

Gene panel list Hannover (47 genes)

ANKRD26, ASXL1 (exon 12-13), ASXL2 (exon 9, 11-13), BCOR, BCORL1, BRAF (exon 12-17), CBL (exon 8-9), CDKN2A, CEBPA, CKIT (exon 1-3, 8-13, 16-18, 20-21), CUX1, DDX41, DNMT3A, ETV6, EZH2, FLT3, GATA1, GATA2, GNAS (exon 1, 8-9), IDH1, IGH2, IKZF1, JAK2 (exon 12-14), KRAS, NF1, NFE2, NPM1, NRAS, PHF6, PIGA, PRPF8, PTEN, PTPN11 (exon 3-4, 7-8, 12-13), RAD21, SAMD9, SAMD9L, SETBP1, SF3B1 (exon 5-6, 13-16, 18, 24), SNC1A, SMC3, SRSF2, STAG2, TET2, TP53, U2AF1, WT1, ZRSR2
